# *In vivo* blood and *ex vivo* brain solid-phase microextraction coupled with LC-MS/MS for mapping unbound cannabidiol, phase I metabolites, and endocannabinoid tone

**DOI:** 10.3389/fphar.2026.1980760

**Published:** 2026-10-26

**Authors:** Agnieszka Mosińska, Magdalena Kaszewska, Katarzyna Sztormowska-Achranowicz, Katarzyna Smarzewska, Katarzyna Owczarek, Katarzyna Woźniczka, Vaclav Trojan, Maciej Miś, Janusz Pawliszyn, Tomasz Bączek, Anna Roszkowska

**Affiliations:** 1 Department of Pharmaceutical Chemistry, Medical University of Gdańsk, Gdańsk, Poland; 2 Department of Pharmacology, Medical University of Gdańsk, Gdańsk, Poland; 3 Clinical Department of Neurosurgery, Wrocław Medical University, Wrocław, Poland; 4 Department of Biochemistry, Medical University of Gdańsk, Gdańsk, Poland; 5 Cannabis Facility, St. Anne’s University Hospital Brno, Brno, Czechia; 6 Department of Natural Drugs, Masaryk University, Brno, Czechia; 7 Department of Chemistry, University of Waterloo, Waterloo, ON, Canada

**Keywords:** 2-arachidonoylglycerol, blood-brain barrier, cannabidiol, endocannabinoid system, neuropharmacokinetics, solid-phase microextraction

## Abstract

**Introduction:**

Evaluating the central nervous system distribution of cannabidiol (CBD) and its dynamic interplay with the endocannabinoid system (ECS) is fundamentally constrained by conventional tissue homogenization, which fails to distinguish pharmacologically active fractions from total pool of analyte. To resolve active biophase kinetics, this study established a minimally invasive solid-phase microextraction (SPME) coupled with LC-MS/MS platform to monitor CBD, its phase I metabolites, and key endocannabinoids directly within circulating blood *in vivo* and intact anatomical brain regions *ex vivo*.

**Methods:**

Male rats received a single oral dose of medicinal cannabis (100 mg/kg CBD). Systemic fractions of analytes were captured *via in vivo* SPME (10-min sampling window) in the circulating blood of living animals. Following sacrifice, *ex vivo* SPME (30-min sampling window) was deployed across four anatomical brain regions (hippocampus, amygdala, cerebellum, and brainstem). SPME-based microextraction was performed across a 1–6 h temporal baseline, and was then cross-validated against traditional solid-liquid extraction (SLE). Analytes were quantified using validated, high-sensitivity liquid chromatography-tandem mass spectrometry (LC-MS/MS) method.

**Results and Discussion:**

*In vivo* blood sampling captured rapid oral absorption of parent CBD, reaching a maximum free systemic concentration (Cmax ≈160 μg/mL) at 2 h post-dose followed by rapid systemic clearance. Among the phase I metabolites, only 6-hydroxy-CBD (6-OH-CBD) was recovered from circulating blood at the 2-h interval at trace levels. Mean systemic 2-arachidonoylglycerol (2-AG) concentrations were higher than baseline at all post-dose time points, with the highest mean value observed at 6 h. *Ex vivo* brain microextraction uncovered marked neuropharmacokinetic heterogeneity. Furthermore, while total tissue SLE detected abundant phase I metabolites with total 7-OH-CBD higher than parent CBD, SPME may reflect limited freely available concentrations and/or strong tissue association of those analytes in brain structures. This exploratory study confirms the technical feasibility of coupling living intravascular extractions with localized regional brain sampling of CBD and endocannabinoids. By demonstrating that CBD concentrations clear dynamically and account for only a minute fraction of total tissue homogenate mass, this methodology-driven proof of concept establishes a more accurate bioanalytical framework for evaluating novel ECS modulators and lipophilic neuropharmacological candidates.

## Graphical Abstract

**Figure:**
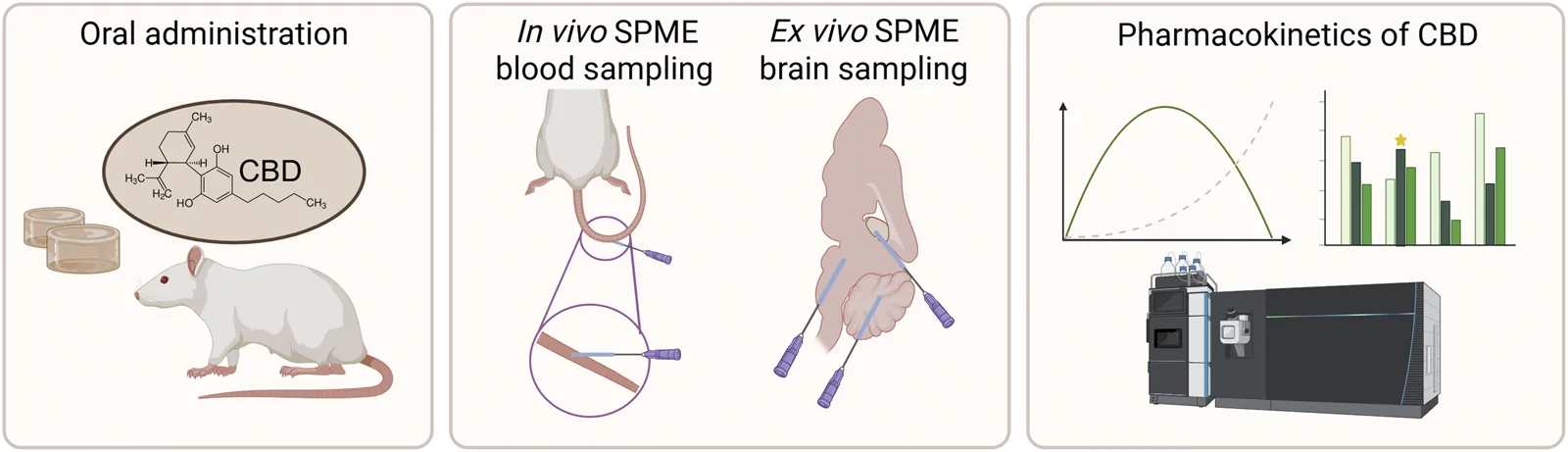

## 1 Introduction

The endocannabinoid system (ECS) has emerged as a cornerstone of modern neuropharmacology, regulating an array of physiological and pathological processes within the central nervous system (CNS), including neuroinflammation, synaptic plasticity, and emotional homeostasis (Aslam et al., 2019; Kruk-Slomka et al., 2017). Among the diverse array of molecules capable of modulating this system, cannabidiol (CBD), the primary non-psychoactive phytocannabinoid derived from *Cannabis sativa* L. has garnered significant attention due to its multifaceted therapeutic profile (Murovec et al., 2022; Schouten et al., 2024). Unlike Δ9-tetrahydrocannabinol (Δ9-THC), CBD acts through complex non-psychoactive pathways, demonstrating profound neuroprotective, anxiolytic, antiepileptic, and anti-inflammatory properties across various preclinical models and clinical cohorts (Sadaka et al., 2021; Schouten et al., 2024). Despite its widespread therapeutic deployment, the clinical use of CBD remains heavily bottlenecked by its unfavorable physicochemical and pharmacokinetic profiles, particularly when administered *via* the oral route (Kaszewska et al., 2025).

Oral ingestion represents the most common and patient-compliant route of drug delivery, however, this route of administration of CBD is plagued by poor and highly erratic systemic bioavailability, primarily driven by intense hepatic first-pass metabolism and low aqueous solubility (Antunes et al., 2023; Yau et al., 2023; Moazen-Zadeh et al., 2024). The metabolism of CBD occurs predominantly in the liver and proceeds in two phases. Phase I is catalyzed by CYP450 enzymes (principally CYP2C19, CYP2C9, CYP3A4), which hydroxylate CBD to active metabolites, i.e., 7-hydroxy-cannabidiol (7-OH-CBD), 6-hydroxy-cannabidiol (6-OH-CBD), and inactive 7-carboxy-cannabidiol (7-COOH-CBD). Phase II encompasses conjugation reactions, primarily glucuronidation mediated by UDP-glucuronosyltransferase, yielding more water-soluble metabolites excreted mainly in urine and, to a lesser extent, in feces. Interindividual variability in CYP450 enzyme expression may significantly influence the pharmacokinetics of CBD and its metabolites, thereby affecting both therapeutic outcomes and the adverse effect profile (Kaszewska et al., 2025). As a highly lipophilic compound, absorbed CBD undergoes rapid and extensive distribution out of the central circulation and into lipid-rich matrices and highly perfused organs, most notably crossing the blood-brain barrier (BBB) to interact with its molecular targets in the CNS (Antunes et al., 2023; Babayeva and Srdanovic, 2024; Martinez Naya et al., 2024). Consequently, although plasma CBD concentrations represent the primary measure of systemic exposure, they do not necessarily reflect either the magnitude or the duration of drug exposure within the brain. Preclinical studies indicate that the relationship between plasma and brain CBD concentrations may vary over time and depends on the route of administration as well as the formulation employed (Deiana et al., 2012; Calapai et al., 2020). Furthermore, available evidence suggests that CBD distribution within the brain is region-specific, with concentrations differing among individual brain structures (Upadhyay et al., 2025). While pharmacokinetic (PK) profiles can be delineated through total blood concentration curves, understanding the distinct regional PKs within specialized brain structures is crucial for predicting localized pharmacodynamic effects and therapeutic outcomes of CBD and its active metabolites (Sadaka et al., 2021). The assessment of regional CBD distribution may reveal spatial differences that cannot be detected in whole-brain homogenates and may provide more detailed and biologically relevant information regarding CBD exposure within individual brain structures. However, studies directly comparing CBD pharmacokinetics across multiple brain regions remain limited (Brookes et al., 2023; Upadhyay et al., 2025).

The ECS is regulated by endogenous lipid mediators, whereas exogenous phytocannabinoids, including CBD, may modulate its activity. One of the principal endocannabinoids is 2-arachidonoylglycerol (2-AG), the most abundant endocannabinoid in the CNS and a highly efficacious full agonist of both CB1 and CB2 cannabinoid receptors (Bedse et al., 2020; Lu and Mackie, 2021; Briand-Mésange et al., 2024). In contrast to 2-AG, CBD exhibits low affinity for the orthosteric binding sites of cannabinoid receptors and is thought to modulate endocannabinoid signaling predominantly through indirect mechanisms, including negative allosteric modulation of the CB1 receptor (Stasiulewicz et al., 2020). Regional changes in 2-AG concentrations in the brain reflect the dynamic balance between local synthesis and degradation, and may therefore provide insight into the functional status of the ECS. Since CBD may indirectly influence endocannabinoid signaling, temporal assessment of regional 2-AG concentrations may complement the evaluation of CBD distribution within the brain and provide insight into accompanying biological responses (Chester et al., 2024).

Traditionally, tracking the brain penetration of CNS therapeutics has relied on bulk tissue homogenization followed by solid-liquid extraction (SLE) or liquid-liquid extraction (Castell et al., 2024). Although SLE remains a valuable analytical benchmark for quantifying total tissue concentrations, it possesses fundamental limitations when interpreting neuropharmacological efficacy. According to the long-standing “free drug hypothesis”, only the unbound fraction of a drug molecule is capable of diffusing to, binding with, and actively modulating its corresponding receptors or enzymes (Cibotaru et al., 2021). For highly lipophilic molecules like CBD, which exhibit extensive non-specific binding to plasma lipoproteins and structural tissue proteins (>95%), total tissue concentrations obtained *via* SLE fail to differentiate between the inert, protein-bound reservoir and the pharmacologically active fraction (Correia et al., 2023; Binova et al., 2025). Furthermore, conventional tissue sampling and sample preparation techniques are susceptible to post-mortem ischemic artifacts and metabolic shifts that can distort the true neurochemical landscape. To overcome these sampling challenges, biocompatible solid-phase microextraction (SPME) has emerged as a powerful, non-depletive sampling platform and an alternative to traditional microdialysis of brain samples (Lendor et al., 2019; Bojko, 2022). Operating as a miniaturized “chemical biopsy”, SPME utilizes a thin, flexible probe coated with a biocompatible polymeric stationary phase that selectively adsorbs small molecules (<1,500 Da) directly from the biological matrix without removing any physical fluid or tissue volume (Bogusiewicz et al., 2021; Roszkowska et al., 2023). Because the extraction process relies on the partitioning between the analyte and the probe coating (extraction phase), SPME measures the active fraction without disrupting the preexisting protein-binding equilibrium (Cibotaru et al., 2021; Jaroch et al., 2021). Furthermore, the immediate isolation and immobilization of analytes on the fiber phase capture the metabolites, preventing enzymatic breakdown by local hydrolases or post-mortem degradative pathways (Riboni et al., 2020; Yu et al., 2022; Woźniczka et al., 2023). While *in vivo* SPME has been validated for tracking endogenous lipid mediators like endocannabinoids in animals, its application for the multi-matrix mapping of exogenous phytocannabinoids remains underexplored (Aslam et al., 2019; Roszkowska et al., 2023; Oliveira et al., 2026).

In this study, we address this knowledge gap by validating a multi-matrix analytical workflow that cross-correlates systemic circulating levels with regional central distribution profiles of CBD and its metabolites along with selected endocannabinoids. Following a single oral administration of CBD to rats, *in vivo* SPME sampling was performed directly in circulating whole blood of living animals using a 10-min exposure window to establish the systemic CBD fraction. To investigate the spatial compartmentalization within the CNS, animals were sacrificed, and *ex vivo* SPME was deployed across four anatomically distinct brain regions (hippocampus, amygdala, cerebellum, and brainstem) to extract localized concentrations of CBD and its metabolites, and also endocannabinoids. Finally, the exact same brain regions were subjected to classical SLE to quantify total tissue concentrations of above-mentioned analytes. CBD and 2-AG concentrations were evaluated over time in the systemic circulation and in four anatomically and functionally distinct brain structures. This approach enabled the assessment of regional differences in the rate and extent of CBD distribution and provided a basis for comparing the temporal relationship between systemic exposure and central tissue disposition in respect to the functioning of the ECS. By evaluating the direct correlation between *in vivo* blood free levels, *ex vivo* brain free profiles, and total regional drug burdens facilitated by optimized and validated LC-MS/MS method, this work establishes a precise framework for evaluating the spatial pharmacokinetics of ECS modulators, directly advancing our ability to evaluate preclinical candidates in neuropharmacology.

## 2 Materials and methods

### 2.1 Chemicals

Chemical standards of CBD and endocannabinoids (AEA, 2-AG, NADA) were purchased from Merck (Darmstadt, Germany). Standards of CBD metabolites, namely 7-OH-CBD, 6-OH-CBD, and 7-COOH-CBD were obtained from Merck (Darmstadt, Germany). Deuterated isotopologue of AEA - AEA-d_11_ was obtained from Cayman Chemical (United States). Deuterated isotopologue of CBD - CBD-d_3_ was obtained from Merck (Darmstadt, Germany). LC-MS-grade methanol (MeOH), acetonitrile (ACN), isopropanol (IPA), formic acid (FA), ammonium formate and chloroform were supplied by Merck (Darmstadt, Germany). Water used in the experiments was purified with a milli-Q system (Molsheim, France). Biocompatible SPME probes with C18 coating, low gelling temperature agarose and bovine serum were supplied by Merck (Darmstadt, Germany). Phosphate-buffered saline (PBS) was obtained from Cayman Chemical (United States). A stock solutions of endocannabinoids, CBD and its metabolites, and also deuterated internal standards (ISs) were prepared in MeOH. Working standard solutions of analytes were prepared by dissolving an appropriate volume of stock solution in the working solutions. A working standard solution of ISs at a concentration of 100 μg/mL was prepared by dissolving an appropriate volume of IS stock solution in MeOH. The stock and working solutions of all analytes were stored at −80 °C.

### 2.2 Animal study

The experiments were performed in accordance with the Guide for the Care and Use of Laboratory Animals issued by the European Parliament, Directive 2010/63/EU, and following approval by the Local Ethical Committee for Animal Experimentation in Bydgoszcz, Poland (21/2024). All experimental procedures were conducted on male Wistar Han rats weighing 200 ± 10 g. The animals were housed under controlled conditions at a temperature of 23 ± 2 °C, a relative humidity of 55 ± 10%, and a constant 12-h light/dark cycle. To determine the pharmacokinetics of the tested compound, the rats were randomly assigned to one of five groups (n = 2–3 per group). The first group, which served as the control group, received plain jelly without CBD or other active ingredients, and was used to establish baseline levels of the analyzed compounds prior to CBD exposure. Control animals were sampled at baseline (0 h). The remaining four groups were assigned to specific time points: 1, 2, 4, and 6 h after CBD administration. The animals were provided with water and standard chow *ad libitum*. For 12 h prior to the experimental procedure, animals were fasted, although they had unlimited access to drinking water. On the day of the experiment, the groups assigned to specific time points were administered a single oral dose of CBD in the form of a jelly at a dose of 100 mg/kg of body weight. Immediately before CBD administration, and subsequently at 1, 2, 4, and 6 h post-administration, *in vivo* SPME sampling of analytes from the blood was performed. The experimental timing was initiated after the entire jelly containing the assigned CBD dose had been consumed (ca. 3 min after administration). Three animals were initially assigned to each time point; however, the final number of evaluable animals was n = 2–3 depending on the time point. In one case, an animal assigned to the 1-h sampling point died before sample collection. In the remaining cases in which n = 2, the reduced sample size resulted from technical issues related to SPME sampling, which prevented obtaining a valid analytical measurement from one animal. For *in vivo* SPME sampling, to induce and maintain anesthesia, each animal was connected to a small animal anesthesia system using an appropriate mixture of isoflurane (induction: 5%; maintenance: 2.5–3%) with oxygen. After the designated 10 min, the SPME probe was removed, and the animal was euthanized (3 rats were sacrificed per time point) to collect brains for *ex vivo* SPME sampling.

### 2.3 Preparation of jellies with CBD for oral administration

Cannabis plant material with a CBD-dominant chemotype (Type III), containing 7.6% CBD used for the preparation of jellies in this study, was obtained from the Cannabis Facility of the International Clinical Research Center (ICRC) at St. Anne’s University Hospital in Brno (FNUSA), Brno, Czech Republic. This facility holds three licenses issued by the State Institute for Drug Control (SÚKL), covering Good Agricultural and Collection Practice (GACP), Good Manufacturing Practice (GMP), and Good Distribution Practice (GDP). The CBD-dominant chemotype was cultivated under controlled conditions (light regime, temperature, humidity, and nutrition) in accordance with the established GACP/GMP procedures. The dried medicinal cannabis raw material was subjected to decarboxylation based on the method of Landa et al., with modifications (Landa et al., 2018). The dried plant material was first weighed into glass jars. Decarboxylation was carried out in a laboratory dryer (SLW 115 STD, POL-EKO, Wodzisław Śląski, Poland) at 121 °C for 60 min. The plant material was then allowed to cool and was stored in tightly sealed glass jars at −18 °C to prevent excessive loss of volatile oils. Prior to jelly preparation, the decarboxylated plant material was ground and homogenized, and its CBD content was verified by high-performance liquid chromatography with ultraviolet detection (HPLC-UV). The CBD content of the decarboxylated plant material was calculated based on data obtained from HPLC-UV analysis, performed in accordance with the European Pharmacopoeia monograph Cannabis Flos (No. 3028) (Council of Europe, 2024). Jellies intended for pharmacokinetic studies in rats were prepared based on the protocol for voluntary oral administration of substances to laboratory animals (Zhang, 2021). To prepare the gel mass, 8 g of food-grade gelatin was weighed and dissolved in 100 mL of distilled water. To the resulting solution, 50 g of glucose was added, and the mixture was placed on a magnetic stirrer with heating until a homogeneous mass was obtained. Decarboxylated hemp plant material was weighed in amounts of 260 mg per jelly, using a watch glass as a weighing vessel. The weighed samples were quantitatively transferred to a mortar and moistened with 3 drops of rapeseed oil, added using a Pasteur pipette. The material was thoroughly mixed with the oil using a pestle until a homogeneous mass was obtained. The jelly mold was pre-coated with a thin layer of rapeseed oil to facilitate subsequent removal of the product. The moistened hemp material was evenly distributed into the cavities of the mold and then covered with the prepared gelatin mass. The jellies were left at room temperature until complete solidification. Once the gel mass had set, the finished jellies were removed from the mold.

### 2.4 *In vivo* blood sampling *via* SPME

To characterize the circulating free (unbound) fraction of CBD and its metabolites in living systems, *in vivo* SPME sampling was performed prior to CBD administration and at the designated time points (1, 2, 4, or 6 h post-dose). The designated sampling time points were calculated from the time at which the entire CBD-containing jelly had been consumed. The SPME probe was introduced into the blood vessel exactly at the respective designated time point (1, 2, 4, or 6 h post-dose), and the 10-min sampling period commenced immediately after probe insertion. SPME probes were autoclaved at 121 °C for 20 min and then were subjected to conditioning in a 1 mL mixture of MeOH/water (50/50, v/v) for 30 min at 600 rpm. Next, SPME probes were rinsed with sterile water and applied for SPME sampling of blood under *in vivo* conditions. Before SPME experiments, animals were anesthetized as described above. Direct puncture of the lateral tail vein was performed to obtain vascular access. Additionally, to minimize pain and procedural discomfort associated with venipuncture, the puncture site was locally anesthetized using a 2% lidocaine-containing gel. A biocompatible SPME probe coated with 10 mm length C18 extraction phase was introduced into the vessel lumen. The SPME probe was exposed directly to circulating blood for exactly 10 min, operating within a pre-equilibrium extraction window to isolate the analytes. Upon completion of the 10-min window, the SPME probe was retracted, immediately removed from the vessel, and gently rinsed with ultra-pure water for 5 s to remove loosely adherent blood cells or plasma proteins. The wiped SPME probes were placed into empty glass vials and stored at −80 °C until subsequent desorption and LC-MS/MS analysis.

### 2.5 *Ex vivo* brain sampling *via* SPME

Immediately following the *in vivo* blood sampling, animals were deeply anesthetized with isoflurane and then euthanized according to a protocol approved by the Local Ethics Committee. After confirming a lack of reflexes, a decapitation (cervical dislocation) was performed, and the skull was rapidly opened, and the entire brain was removed. To determine localized free (unbound) concentrations without cellular matrix distortion, *ex vivo* SPME was immediately deployed in the freshly isolated brain regions. For the sampling of intact brain structures, autoclaved and preconditioned 5 mm length C18 probes were introduced into the amygdalae, cerebellum and hippocampus, and 10 mm length C18 probes were introduced into the brainstem for 30 min static extraction at room temperature. No vascular perfusion was performed prior to tissue collection, as an additional perfusion step could alter the distribution and local availability of analytes and therefore differ from the physiological conditions intended to be captured at the time of sampling. After the extractions had been completed, the probes were removed from the brain tissue, rinsed in ultra-pure water for 5 s to remove any loosely adhered biological matter, wiped with a Kimwipe, and placed in empty glass vials. The probes were stored in the empty vials at −80 °C until subsequent desorption and LC-MS/MS analysis.

### 2.6 LC-MS/MS analysis

On the day of instrumental analysis, the SPME probes were desorbed into 50 μL (for 5 mm length coating) or 100 μL (for 10 mm length coating) mixture of MeOH/IPA (50/50, *v/v*) in silanized inserts for 30 min at 600 rpm. Next, 1 μL of AEA-d11 and CBD-d_3_ were added to the desorption solution to obtain final concentration of 100 ng/mL. For SLE, dry residue was dissolved in 100 µL of MeOH/IPA mixture (50:50, *v/v*), vortexed for 30 s, centrifuged (3,400 rpm, 10 min, 4 °C), and the supernatant was transferred into silanized inserts. Next, all extracts were injected into the LC-MS/MS system for targeted analysis of CBD, its metabolites, and endocannabinoids. Chromatographic separation and mass spectrometric detection were performed using a custom-optimized LC system coupled to a triple quadrupole mass spectrometer (LC-MS/MS) (Agilent 1,290 coupled to an AB Sciex Triple Quad 6500+). Separation of target analytes was achieved using a Agilent Poroshell 120 EC-C18 chromatographic column (2.1 mm × 100 mm, 2.7 μm particle size) maintained at temperature of 40 °C. Autosampler (Nexera Autosampler XR SIL-40 XR) temperature was set at 5 °C. Elution was carried out using a binary gradient system consisting of mobile phase A (water with 0.1% FA) and mobile phase B (ACN with 0.1% FA). The gradient elution program was optimized as follows: 0–1.5 min at 35% B; 1.5–6.0 min, 35%–100% B; 6.1–7.8 min 35% B, from 7.8 min–column equilibration. Total analysis time was 7.8 min. The flow rate increasing from 0.3 to 0.4 mL/min in the first minute. Injection volume was 5 µL. The MS operated utilizing an electrospray ionization (ESI) source configured in positive and negative ion modes. Quantitative detection was executed using multiple reaction monitoring (MRM) modes. Target precursor/product ion transitions, collision energies, and declustering potentials were custom-optimized for each analyte (Supplementary Table S1). For 2-AG, the analytical signal was calculated as the sum of the chromatographic peak areas corresponding to 2-AG and its 1-AG isomer. Thus, the reported 2-AG concentrations represent the combined 2-AG/1-AG signal and do not distinguish between the individual isomers. Source parameters, including gas temperatures, gas flows, and capillary voltages, were optimized to maximize signal intensities for both trace endogenous markers and high-abundance systemic metabolites within a single analytical injection. The MS parameters were as follows: nebulizing gas flow rate: 3 L/min, heating gas flow rate: 10 L/min, interface temperature: 300 °C, desolvation line temperature: 250 °C, heating block temperature: 400 °C, drying gas flow rate: 7 L/min, collision gas: 9 kPa. Spray voltage in positive ionization mode was 5500 V, and −4500 V in negative ionization mode.

### 2.7 SPME-LC-MS/MS method validation

Quantitative method validation was conducted in accordance with bioanalytical method validation guidelines, evaluating linearity, limits of detection (LOD), and limits of quantification (LOQ) (Table 1). Matrix-matched calibration curves were constructed by spiking the respective surrogate matrices (agarose gel, serum) with working solutions of studied analytes. For serum samples, a calibration curve was 1–75 μg/mL for CBD, 1–500 ng/mL for 2-AG, 10–500 ng/mL for 7-OH-CBD, 6-OH-CBD, AEA and NADA, and 50–500 ng/mL for 7-COOH-CBD. For agarose gel samples, a calibration curve was 1–1,000 ng/g for 2-AG, 10–1,000 ng/g for CBD, AEA and NADA, 10–500 ng/g for 7-OH-CBD and 6-OH-CBD, and 25–500 ng/g for 7-COOH-CBD. A mix of ISs (CBD-d_3_ and AEA-d_11_) was added at a fixed concentration (100 ng/mL) to desorption mixture for all calibration curve samples and experimental samples. Linearity was assessed by calculating the coefficient of determination (r^2^) using a weighted linear regression model. The limit of detection (LOD) and limit of quantification (LOQ) were defined as the concentrations yielding signal-to-noise (S/N) ratios of approximately 3 and 10, respectively. Intra-day and inter-day precision and accuracy were evaluated by analyzing quality control (QC) samples at low (LQC), medium (MQC), and high (HQC) concentration levels across three independent validation runs in range of 200 ng, 400 ng and 800 ng (for 2-AG and CBD) and 50 ng, 200 ng and 400 ng (for 7-OH-CBD, 6-OH-CBD, 7-COOH-CBD, AEA, and NADA). Details for SPME method optimization and SLE-LC-MS/MS method validation are presented in the Supplementary Materials.

**TABLE 1:** Linearity parameters, LOQ and LOD for all analyzed compounds.

Analyte	Range	Linear regression	r^2^	LOD	LOQ
Serum				(ng/mL)	(ng/mL)
CBD	1–75 μg/mL	y = 1E+06x + 2E+06	0.99	1.0	10
7-OH-CBD	10–500 ng/mL	y = 7920.3x - 15911	0.99	2.0	10
6-OH-CBD	10–500 ng/mL	y = 5400x - 4060.4	0.99	2.0	10
7-COOH-CBD	50–500 ng/mL	y = 1956.1x + 30112	0.98	10	50
2-AG	10–500 ng/mL	y = 1,082.4x + 35339	0.99	2.0	10
AEA	10–500 ng/mL	y = 912.68x + 20396	0.99	2.0	10
NADA	10–500 ng/mL	y = 2866.8x - 22103	0.99	2.0	10
Agarose gel				(ng/g of tissue)	(ng/g of tissue)
CBD	10–1,000 ng/g	y = 7094.6x - 55439	0.999	1.0	10
7-OH-CBD	10–500 ng/g	y = 1,311.8x + 107499	0.99	2.0	10
6-OH-CBD	10–500 ng/g	y = 3,804.1x + 76633	0.98	2.0	10
7-COOH-CBD	25–500 ng/g	y = 9149x - 112650	0.98	5	25
2-AG	10–1,000 ng/g	y = 5017.8x - 205078	0.99	2.0	10
AEA	10–1,000 ng/g	y = 2039.9x + 8542.7	0.98	2.0	10
NADA	10–1,000 ng/g	y = 3,122.3x - 125046	0.97	2.0	10

### 2.8 Data analysis and statistical evaluation

Chromatographic data processing and peak integration were handled using SCIEX OS software (SCIEX, Toronto, Canada). Three animals were initially assigned to each sampling time point. Samples that did not meet predefined analytical quality criteria (e.g., unsatisfactory chromatographic signal quality or interference) were excluded from further analysis. Consequently, the final number of biological replicates varied between 2 and 3 across sampling time points. Pharmacokinetic (PK) analysis was performed using the naive pooling approach, in which the mean CBD concentration at each sampling time point was calculated from data obtained from 2 to 3 animals. Because brain tissue collection was terminal, individual concentration–time profiles could not be obtained from the same animals. PK parameters (Cmax, Tmax, AUC_0–6h_) were therefore estimated descriptively from the mean concentration–time profiles. AUC_0–6h_ was calculated using the linear trapezoidal method without extrapolation beyond the last sampling time point. These parameters were considered exploratory and descriptive, and were not used for formal statistical comparisons between brain regions or for assessment of inter-individual variability in PK exposure. Statistical analyses and graphical presentations were performed using GraphPad Prism version 11.0.2 (GraphPad Software, San Diego, CA, United States). Owing to the small sample size per group, non-parametric tests were applied. Temporal changes in CBD and 2-AG concentrations were analyzed separately for each brain region using the Kruskal–Wallis test followed by Dunn’s multiple comparisons test. Data are presented as mean ± SD. A p value <0.05 was considered statistically significant.

## 3 Results and discussion

### 3.1 SPME-LC-MS/MS methodology

This study aimed to apply a minimally invasive SPME sampling for simultaneous analysis of CBD and its metabolites (6-OH-CBD, 7-OH-CBD, 7-COOH-CBD), and also endocannabinoids (AEA, 2-AG, NADA) in biological samples (blood, brain). For the first time, CBD pharmacokinetics was monitored under *in vivo* conditions in a living organism. The initial stage of this work included optimization of analytical conditions, namely LC-MS/MS and selected SPME parameters, enabling the most efficient extraction for all analytes from complex matrices (Supplementary Material). In addition, the impact of sampling temperature on stability of analyzed compounds was tested, and the results showed no significant differences in respect to extraction efficiency of analyzed compounds between two tested sampling temperatures, i.e. 20 °C ± 2 °C (room temperature) and 35 °C ± 2 °C (physiological temperature) (Supplementary Figure S1), hence further studies concerning method validation were performed at room temperature. Also, the immediate removal and freezing of the SPME probes after sampling were applied to minimize potential post-sampling degradation or isomerization of analytes, such as 2-AG prior to desorption and LC-MS/MS analysis.

In the next stage, the optimized SPME-LC-MS/MS protocol was applied for the precise analysis of target compounds in real samples during *in vivo* (blood) and *ex vivo* (brain) sampling of rats, who received single oral dose of medicinal cannabis (CBD-dominant chemotype) at CBD concentration of 100 mg/kg of body weight. Validation experiments were conducted on the proposed SPME-LC-MS/MS protocol to confirm its suitability for the monitoring of studied compounds in real biological matrices. Due to endogenous nature of endocannabinoids and strong binding properties of all studied analytes to tissue components, the analyses were performed in 4% low temperature agarose gel and artificial serum with the use of silanized glassware (Roszkowska et al., 2023; Oliveira et al., 2026). The agarose gel was used to reproduce the restricted diffusion and mass-transfer resistance expected in a semi-solid brain matrix. For serum sampling, the proposed method’s linearity was tested by analyzing five series of calibration samples enriched with working standard solutions of each analyte at the following concentrations: 1–75 μg/mL for CBD, and 1–500 ng/mL for all remaining analytes. The ISs (AEA-d_11_ and CBD-d_3_) were spiked into the desorption solvent after the desorption was completed to obtain final concentration of 100 ng/mL. It must be emphasized that the *in vivo* sampling of blood and *ex vivo* sampling of intact brain tissues possessed inherent limitations that preclude the use of IS (Roszkowska et al., 2018). Hence, IS was only added to the sample extracts, thus correcting for instrumental analysis, but not for the extraction process. As shown in Table 1, the mean correlation coefficient (r^2^) was >0.99 for all analytes in serum samples, and >0.97 for all analytes in agarose gel, indicating good linearity over their respective concentration ranges. The method’s LOD was defined as the concentration of each analyte in brain/blood samples at which a signal-to-noise ratio (S/N) of 3:1 was achieved (n = 6). In this research, the LODs for serum and agarose samples were 1 ng/mL for CBD and 2 ng/mL (ng/g of solid tissue) for remaining analytes, except for 7-COOH-CBD for which LODs for serum and agarose gel were equal to 10 ng/mL and 5 ng/g, respectively. The LOQ was defined as the lowest concentration of each analyte in the sample matrix yielding a S/N of 10:1, and precision and accuracy <15% and between 80% and 120%, respectively. Sample preparation and LC-MS/MS analysis of analytes in brain and blood samples were conducted according to the protocols described in Materials and Methods section, with the analyte concentrations being calculated based on the corresponding calibration curve. Carry-over was evaluated by applying the optimized SPME-LC-MS/MS method to analyze samples containing analytes at concentrations of 100 ng/mL and blank samples without analytes, each in triplicates. The obtained data indicate that carry-over was below 2%, which did not affect the method’s ability to quantify each analyte in real biological samples.

### 3.2 *In vivo* SPME blood sampling

*In vivo* SPME sampling within the circulating blood of living, anaesthetized rats revealed distinct, time-dependent kinetic profiles for CBD following a single oral dose. Within the systemic circulation, CBD exhibits a very high plasma protein binding capacity (Moazen-Zadeh et al., 2024). By implementing a 10-min microextraction window, the sampling platform successfully isolated systemic concentrations of CBD from rat’s blood. The level of this parent phytocannabinoid rose rapidly after the first hour, achieving a maximum concentration (around 160 μg/mL) at approximately 2 h post-administration (Figure 1). This fast absorption phase was followed by a marked decline in circulating CBD concentrations at 4 and 6 h, suggesting rapid distribution into highly perfused tissues, including the brain, together with ongoing hepatic metabolism and systemic elimination (Martinez Naya et al., 2024). The kinetic cascade of CBD is governed by intense hepatic first-pass metabolism, primarily mediated by cytochrome P450 enzymes (such as the CYP2C and CYP3A subfamilies in rodents), which rapidly biotransform the parent molecule into its primary hydroxylated and carboxylated products. CBD as a highly lipophilic molecule undergoes distribution into well-perfused organs such as the brain, heart and lungs (Kicman and Toczek, 2020; Child and Tallon, 2022; Brookes et al., 2023; Martinez Naya et al., 2023). The rapid systemic clearance of parent CBD highlights the profound pharmacokinetic challenges associated with the oral delivery of plant cannabinoids (Antunes et al., 2023).

**FIGURE 1:**
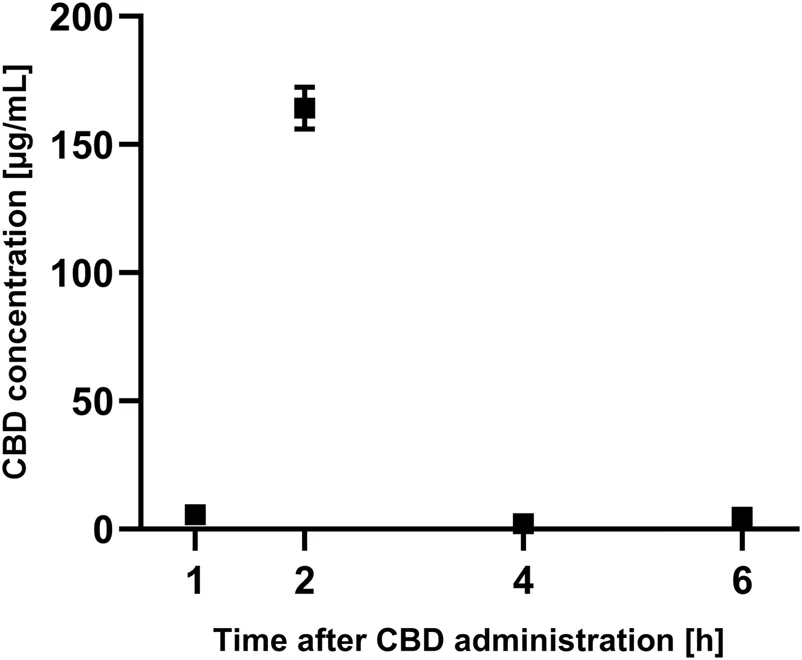
Profiling of CBD concentrations in rat’s blood over 6 h post CBD administration using *in vivo* SPME sampling.

Interestingly, the 10-min *in vivo* blood extraction protocol facilitated the quantification of only one primary pharmacologically active metabolite, namely 6-OH-CBD, which was detected exclusively at the 2-h post-dose interval at trace levels. The other major phase I biotransformation products, i.e., 7-OH-CBD and 7-COOH-CBD remained completely below the detection limits in their unbound forms in systemic circulation throughout the entire 6-h time course. The lack of peripheral detection of those metabolites may suggests that the systemic generation of these metabolites is strictly rate-limited by parent drug availability, keeping their absolute free circulating concentrations significantly lower than those of parent CBD, and also may be related to fast organ distribution of those metabolites.

Simultaneously, the 10-min *in vivo* blood SPME protocol successfully captured changes in circulating endocannabinoid tone, tracking the concentrations of 2-AG in living rats (Figure 2). Animals treated with a single oral dose of CBD exhibited elevated systemic levels of 2-AG relative to vehicle-treated control cohorts. This circulating 2-AG pool increased progressively over the temporal baseline, reaching its maximum concentration at the terminal 6-h post-dose mark. The mean blood concentration of 2-AG in the control group was 34.45 ng/mL. Following CBD administration, serum 2-AG concentrations increased by approximately 178%, 172%, 201%, and 424% at 1, 2, 4, and 6 h, respectively, compared with the control value (Supplementary Table S2). While this trend indicates systemic endocannabinoid upregulation, the preliminary nature of these findings must be interpreted within the context of the small exploratory sample size (n = 2–3 rats per time point) utilized for this proof-of-concept validation. The gradual increase in blood 2-AG concentration, peaking at 6 h, suggests a generalized, but delayed response of the ECS to CBD administration. This profile corroborates the well-documented fact that CBD does not directly activate cannabinoid receptors (CB1 and CB2), but rather acts as an indirect modulator of this system (Castillo-Arellano et al., 2023; Kruk-Slomka et al., 2025). Interestingly, other endocannabinoids have not been detected during 10 min blood sampling period.

**FIGURE 2:**
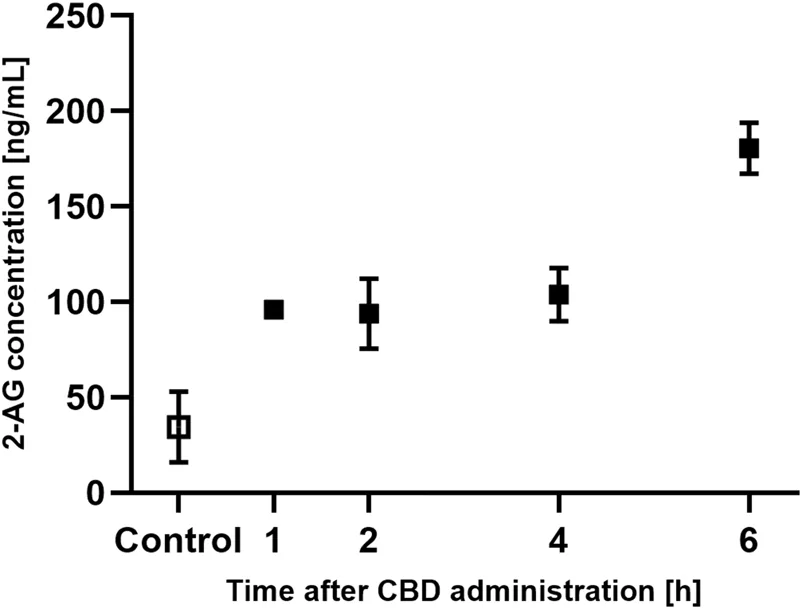
Profiling of 2-AG concentrations in rat’s blood in control group and in rats over 6 h post CBD administration using *in vivo* SPME sampling.

### 3.3 *Ex vivo* SPME brain sampling of CBD and its metabolites

The brain comprises anatomically and functionally distinct regions that differ in neuronal organization, vascularization, neurovascular unit characteristics, lipid composition, and endocannabinoid signaling (Yau et al., 2023; Pfau et al., 2024; Schouten et al., 2024; Blanchette et al., 2025). Such regional heterogeneity may influence the delivery, BBB penetration, local distribution, and retention of lipophilic compounds, thereby contributing to regional differences in CBD pharmacokinetics. The SPME-based sampling highlighted pronounced regional variations and selective BBB partitioning across the central nervous system over the 6-h timeline. SPME was performed immediately after animal sacrifice (Bogusiewicz et al., 2021). A 30-min *ex vivo* SPME static sampling of intact brain structures was performed utilizing custom probe lengths, namely a 5 mm coating for the hippocampus, amygdala, and cerebellum, and a 10 mm coating for the brainstem. Clear neuropharmacokinetic differences in the SPME-accessible fraction of parent CBD were detected between distinct central structures. The hippocampus exhibited higher observed levels of parent CBD compared with other brain regions sampled, suggesting regional heterogeneity in CBD distribution across the blood–brain interface (Table 2). Furthermore, distinct temporal shifts were recorded between these structures, indicating that while CBD fraction achieved its maximum concentration at the 2-h post-dose mark within the amygdala, cerebellum (Supplementary Figure S2), and brainstem (data not shown), the peak concentration within the hippocampus was delayed, reaching its maximum at 4 h post-administration. Strikingly, no phase I CBD metabolites were extracted from any of the studied brain structures *via* the SPME probes, with the exception of 7-OH-CBD that was detected in the hippocampus and the amygdala of one rat at 4 h time-point. Further SLE analysis revealed that this one rat represented extreme levels of CBD and its metabolites in all brain tissues sampled, confirming inter-individual differences in metabolism of CBD and BBB penetration among rats. The absence of CBD metabolites in the brain structures may be driven by distinct phenomena, such as high non-specific binding and immobilization of these molecules to structural lipophilic elements within the brain tissue matrix, and/or localized free concentrations falling below the chemical enrichment capacity of the SPME coating.

**TABLE 2:** Mean CBD concentrations (ng/g) in rat brain following oral administration of a single dose of CBD (100 mg/kg).

Brain region	Time (h)				p
	1	2	4	6	
Amygdala	123.12 ± 19.49 (n = 2)	140.83 ± 11.87 (n = 3)	94.37 ± 15.62 (n = 2)	78.59 ± 43.59 (n = 3)	0.0792
Cerebellum	69.30 ± 5.03 (n = 2)	157.80 ± 10.68 (n = 3)	112.46 ± 9.1 (n = 3)	64.18 ± 16.08 (n = 3)	0.0018
Hippocampus	140.67 ± 75.80 (n = 2)	182.66 ± 61.17 (n = 2)	411.03 ± 90.66 (n = 3)	44.23 ± 9.63 (n = 3)	0.0014

Each value represents the mean ± SD, n = 2–3 animals per time point. Kruskal–Wallis tests followed by Dunn’s multiple comparisons tests were performed separately for each brain region. Significant pairwise differences were detected for the cerebellum (2 vs. 6 h: H = 8.803, overall p = 0.0018; Dunn’s p = 0.0406) and hippocampus (4 vs. 6 h: H = 8.127, overall p = 0.0014; Dunn’s p = 0.0278). No significant overall difference was detected in the amygdala (H = 6.036, p = 0.0792).

#### 3.3.1 Pharmacokinetic (PK) profile and distribution of CBD

Following a single oral administration of CBD (100 mg/kg), this phytocannabinoid was detected in all analyzed brain regions, including the amygdala, cerebellum, and hippocampus, at each evaluated time point between 1 and 6 h after dosing (Table 2; Supplementary Figure S2). The concentration–time profiles of CBD differed markedly among the examined brain regions. In the amygdala, the mean CBD concentration was 123.12 ± 19.49 ng/g at 1 h and reached a maximum (Cmax) of 140.83 ± 11.87 ng/g at 2 h (Table 3). Thereafter, CBD concentrations gradually declined, reaching a value that was 44% lower than Cmax at 6 h. Nevertheless, the observed differences between time points were not statistically significant (Kruskal–Wallis test: H = 6.036; p = 0.0792). In the cerebellum, CBD concentrations also peaked at 2 h (157.80 ± 10.68 ng/g), followed by a progressive decline to the lowest concentration observed at 6 h (64.18 ± 16.08 ng/g), corresponding to a 59% reduction relative to Cmax. A significant effect of time on CBD concentration was detected by the Kruskal–Wallis test (H = 8.803; p = 0.0018). Post hoc analysis using Dunn’s multiple comparisons test demonstrated a significant difference between the 2- and 6-h time points (p = 0.0406). In contrast, the hippocampus exhibited a distinct PK profile. The highest CBD concentration was observed 4 h after administration, reaching 411.03 ± 90.66 ng/g, which was approximately 2.9-fold higher than the peak concentration measured in the amygdala and 2.6-fold higher than that in the cerebellum. By 6 h, CBD concentrations had declined to 44.23 ± 9.63 ng/g, representing an 89% decrease from the peak value. Significant differences among time points were identified (Kruskal–Wallis test: H = 8.127; p = 0.0014), and *post hoc* analysis revealed a significant difference between the 4- and 6-h time points (p = 0.0278). The AUC_0–6_, calculated using the linear trapezoidal method from the composite mean concentration–time profile, was numerically higher in the hippocampus (1,280.95 ng/g·h) than in the amygdala (601.70 ng/g·h) and cerebellum (595.09 ng/g·h) (Table 3). Descriptively, the hippocampal AUC_0–6_ was approximately 2.1-fold and 2.2-fold higher than the corresponding values in the amygdala and cerebellum, respectively. To further compare regional CBD exposure relative to systemic availability, the ratio of brain AUC_0–6_ to serum AUC_0–6_ was calculated (Table 4). The numerically highest brain-to-serum AUC_0–6_ ratio was observed for the hippocampus (0.00492 mL/g), whereas lower and nearly identical values were obtained for the amygdala (0.00231 mL/g) and cerebellum (0.00228 mL/g). Taken together, these descriptive findings suggest a distinct regional CBD concentration–time profile in the hippocampus, characterized by a delayed T_max_ and a numerically greater AUC_0–6_ compared with the amygdala and cerebellum.

**TABLE 3:** Pharmacokinetic profile of CBD in three selected brain regions after a single oral administration of CBD at a dose of 100 mg/kg in rat.

Parameter	Amygdala	Cerebellum	Hippocampus
Cmax [ng/g]	140.83	157.8	411.03
Tmax [h]	2	2	4
AUC_0-6_ [ng/g·h]	601.7	595.09	1,280.95

Parameters were derived from the composite mean concentration–time profiles using naive pooling of mean concentrations. For AUC_0–6_, the pre-dose CBD, concentration was assumed to be 0 ng/g. AUC_0–6_ values were calculated using the linear trapezoidal method.

**TABLE 4:** AUC_0-6_ ratio of CBD in plasma and individual brain regions in rats following a single oral dose of CBD (100 mg/kg).

Brain region	AUC_*brain*_/AUC_*plasma*_ [mL/g]
Amygdala	0.00231
Cerebellum	0.00228
Hippocampus	0.00492

AUC ratios were calculated from mean concentration-time profiles.

Overall, SPME sampling facilitated successful detection of CBD in all examined brain regions of rats treated with single oral dose of medicinal cannabis containing CBD (Supplementary Figure S2). The presence of CBD as early as 1 h after administration in the amygdala, cerebellum, and hippocampus confirms its ability to cross the BBB. This observation is consistent with the highly lipophilic nature of CBD and previous rodent studies demonstrating measurable concentrations of CBD in brain tissue following oral administration (Schwotzer et al., 2023; Schouten et al., 2024; Binova et al., 2025). Deiana et al. demonstrated that oral administration of CBD (120 mg/kg) in rats resulted in greater brain exposure than intraperitoneal administration under the experimental conditions used. Furthermore, the temporal profile of the behavioral effects of CBD closely paralleled its pharmacokinetic profile in the brain (Deiana et al., 2012). In the present study, the Tmax of 2 h observed in both the amygdala and cerebellum is consistent with previous PK studies in rodents, in which peak brain concentrations of CBD generally occurred between 1 and 2 h after oral administration. However, it should be emphasized that Tmax is influenced by several factors, including dose, formulation, lipid vehicle, feeding status (fed vs. fasted), and route of administration (Yau et al., 2023; Binova et al., 2025). Oral formulation is of particular importance because CBD exhibits low and highly variable oral bioavailability owing to its poor aqueous solubility and extensive first-pass metabolism (Moazen-Zadeh et al., 2024).

### 3.4 *Ex vivo* SPME brain sampling of endocannabinoids

Among the primary endocannabinoids, only 2-AG was detectable in all brain regions sampled *via* SPME probes. 2-AG is one of the principal endocannabinoids present in the CNS. In the brain, it is synthesized “on demand,” primarily by DAGLα, whereas DAGLβ appears to play a more prominent role in specific cell populations and non-neuronal tissues. Once synthesized in the postsynaptic neuron, 2-AG acts predominantly as a short-range retrograde messenger (Bedse et al., 2020). Brain concentrations of 2-AG are considerably higher than those of AEA, although these two endocannabinoids differ substantially in their biosynthetic pathways, cellular localization, and mechanisms of regulation (Lu and Mackie, 2016; Araque et al., 2017; Zou and Kumar, 2018).

CBD is a known competitive inhibitor of fatty acid amide hydrolase (FAAH), the primary intracellular enzyme responsible for AEA catabolism (Cásedas et al., 2024). Conversely, 2-AG is hydrolyzed primarily by monoacylglycerol lipase (MAGL) rather than FAAH, which may contribute to differences in the regulation of these two endocannabinoids. Following oral CBD administration, *ex vivo* SPME revealed region-dependent temporal patterns in the SPME-accessible 2-AG fraction across the 1–6 h post-dose window (Table 5; Supplementary Figure S3). However, different descriptive temporal patterns in mean 2-AG concentrations were observed in the remaining brain structures. In the amygdala, CBD administration resulted in lower mean 2-AG concentrations at all time points assessed compared to the control group. At 1 and 2 h, the mean concentrations were approximately 15.7% and 28% lower than control, respectively, whereas at 4 and 6 h they remained only slightly below the baseline value. However, no statistically significant differences were detected among the analyzed groups (Kruskal–Wallis test: H = 3.600, p = 0.5704). Mean 2-AG concentrations in the amygdala (Supplementary Figure S3) and brain stem (data not shown) remained numerically close to or below control values across the entire 6-h time course, with no statistically significant overall differences among the analyzed groups. These findings should therefore be interpreted as an absence of statistically detectable temporal changes rather than evidence of definitive regional differences. The cerebellum exhibited an early numerical decline in mean 2-AG concentrations during the first 1–2 h post-dose, followed by recovery toward the control value, but the overall differences did not reach statistical significance. At 2 h, the mean 2-AG concentration remained approximately 36% below control, whereas at 4 h it returned to a value comparable to baseline and declined slightly at 6 h. Despite these numerical differences, the overall Kruskal–Wallis test did not reach statistical significance (H = 7.091, p = 0.0794). In the hippocampus, the 2-AG concentration at 1 h (159.28 ± 74.96 ng/g) was comparable to that of the control group, and a slight decrease was observed at 2 h. In contrast, the hippocampus showed numerically higher mean 2-AG concentrations at 4 and 6 h, with the highest mean value observed at 6 h. These values were approximately 88.0% and 122.9% higher than the corresponding control concentration, respectively. The overall Kruskal–Wallis test indicated a statistically significant difference among the analyzed groups (H = 8.590, p = 0.0227), however, Dunn’s *post hoc* multiple-comparison test did not identify statistically significant differences between individual time points. Accordingly, the temporal profile observed in the hippocampus should be interpreted cautiously and does not establish a significant increase at any specific post-dose time point. The observed hippocampal temporal pattern may result from its unique properties, including high synaptic plasticity, high CB1 receptor density, and significant sensitivity to endocannabinoid system modulation (Somogyi et al., 2025). Changes in neuronal and glial cell activity affecting 2-AG metabolic pathways could potentially contribute to this temporal profile, although the present study does not allow the underlying mechanism to be established. These descriptive, region-dependent temporal patterns in the SPME-accessible 2-AG fraction suggest that the relationship between CBD exposure and central endocannabinoid signaling may differ among brain regions and warrants further investigation in larger cohorts. CB1 receptors are found throughout the brain and mediate the central effects of cannabinoids, and have been identified in the CNS and in high concentrations in regions such as the prefrontal cortex, hippocampus, cerebral cortex, striatum and cerebellum (Tao et al., 2020). Lower expression can be observed in the hypothalamus, periaqueductal gray and basolateral amygdala (Lazenka et al., 2013). Comparing the pharmacokinetics of CBD with the dynamics of 2-AG changes in the hippocampus revealed that the maximum mean CBD concentration in this structure occurred at 4 h. At the same time point, mean 2-AG concentrations began to increase numerically, reaching their highest mean value at 6 hours—at a time when the mean CBD concentration was already declining. This finding may indicate a delayed response of the ECS, potentially associated with the earlier exposure of the hippocampus to CBD through alterations in neuronal activity, modulation of glutamatergic and GABAergic neurotransmission, changes in the synthesis or degradation of 2-AG, or compensatory activation of endocannabinoid signaling pathways (Schurman et al., 2019; Bedse et al., 2020). However, these interpretations remain speculative because the present study did not assess the expression or activity of enzymes involved in 2-AG metabolism, nor did it evaluate CB1 receptor activation. It should also be considered that measurements performed in homogenates of entire brain structures represent an average signal derived from multiple neuronal and glial cell populations with distinct metabolic and synaptic activities. Consequently, such analyses may mask localized and even opposing changes occurring within individual neuronal circuits (Scheyer et al., 2023). Such region- and time-specific responses are consistent with the current understanding of the ECS, according to which 2-AG is synthesized locally in an activity-dependent manner within specific neuronal populations and synaptic circuits. Consequently, 2-AG signaling may differ not only between brain regions but also among individual neuronal populations and synapse types within the same structure (Lu and Mackie, 2016; Araque et al., 2017; Scheyer et al., 2023). It is also important to note that changes in total tissue 2-AG concentration are not necessarily directly proportional to the strength of CB1 receptor-mediated signaling (Di Marzo and Piscitelli, 2015; Lu and Mackie, 2016; Scheyer et al., 2023).

**TABLE 5:** Mean 2-AG concentrations (ng/g) in rat brain following oral administration of a single dose of CBD (100 mg/kg).

Brain region	Time (h)					p
	Control	1	2	4	6	
Amygdala	149.39 ± 20.05 (n = 3)	125.98 ± 41.12 (n = 2)	107.09 ± 5.21 (n = 3)	141.22 ± 9.21 (n = 2)	134.03 ± 44.43 (n = 3)	0.5704
Cerebellum	320.26 ± 113.02 (n = 3)	111.22 ± 5.12 (n = 2)	204.84 ± 46.64 (n = 3)	316.45 ± 107.70 (n = 3)	268.60 ± 25.05 (n = 3)	0.0794
Hippocampus	149.86 ± 19.97 (n = 3)	159.28 ± 74.96 (n = 2)	129.62 ± 14.62 (n = 2)	281.80 ± 22.25 (n = 3)	334.04 ± 62.8 (n = 3)	0.0227

Each value represents the mean ± SD, n = 2–3 animals per time point. Kruskal–Wallis statistics were H = 3.600, p = 0.5704 for the amygdala; H = 7.091, p = 0.0794 for the cerebellum; and H = 8.590, p = 0.0227 for the hippocampus. Although the overall Kruskal–Wallis test was significant for the hippocampus, Dunn’s multiple comparisons test did not identify statistically significant pairwise differences between individual time points.

### 3.5 Cross-correlation of free vs. total brain tissue burden (*ex vivo* SPME vs. SLE)

In addition to SPME sampling, the SLE technique was also applied in this study, and its linearity parameters were determined (Supplementary Table S3). SLE was performed on the same brain structures as for SPME sampling, and the results were then compared with those obtained using SPME. Cross-correlation between *ex vivo* SPME and traditional SLE across brain regions sampled revealed a profound divergence in spatial pharmacokinetics of CBD and its main metabolites. To establish a direct comparison at the predicted peak absorption window, classical SLE was performed on exactly the same tissue samples harvested at each analyzed time-point to contrast total regional drug accumulation against free fractions captured *via* minimally invasive microextraction of intact brain structures.

Following SLE, the regional distribution and concentration-time profiles of CBD and three of its primary metabolites revealed distinct pharmacokinetic behaviors across the studied brain structures over a 6-h period post CBD administration (Supplementary Table S4). Parent CBD demonstrated rapid, but highly region-specific uptake and elimination kinetics. Across most analyzed structures, including the amygdala, hippocampus, and brain stem, CBD concentrations peaked at 2 h post-administration, with the highest concentration observed in the amygdala (4.441 ± 0.06 μg/g of tissue) followed by the brain stem (3.086 ± 0.29 μg/g of tissue). In contrast, the cerebellum exhibited a delayed accumulation of CBD, reaching its peak concentration of 2.260 ± 0.01 μg/g of tissue at the 4-h mark. For all evaluated brain regions, parent CBD cleared rapidly and fell close to the limit of detection by 6 h. The primary active metabolite, 7-OH-CBD, accumulated higher concentrations than parent CBD across all brain regions and exhibited a delayed peak. Concentrations of 7-OH-CBD steadily increased to peak at 4 h in all structures, with the highest concentration occurring in the brain stem (Supplementary Table S4). By 6 h, 7-OH-CBD had cleared to undetectable levels in almost all regions, with the sole exception of the cerebellum, which retained a trace concentrations. The minor hydroxylated metabolite, 6-OH-CBD, displayed a highly uniform distribution and a more sustained pharmacokinetic profile. Peak concentrations of 6-OH-CBD were reached at 2 h in the amygdala, hippocampus, and cerebellum, while the brain stem experienced a delayed peak at 4 h. Unlike CBD and 7-OH-CBD, 6-OH-CBD remained consistently detectable across all four brain structures at the 6-h interval, indicating a slower rate of elimination. Lastly, the terminal inactive metabolite, 7-COOH-CBD, was detected in the lowest concentrations of all the monitored analytes. Peak concentrations of 7-COOH-CBD generally occurred between 2 and 4 h, with the highest concentration observed in the hippocampus at 2 h (0.642 ± 0.34 μg/g of tissue), while the amygdala, cerebellum, and brain stem all peaked at 4 h. At the 6-h mark, 7-COOH-CBD remained detectable in the amygdala and cerebellum, but was cleared below detection limits in both the hippocampus and the brain stem. Overall, these findings demonstrate that parent CBD is rapidly metabolized *in vivo*, resulting in the accumulation of its active metabolite 7-OH-CBD at levels 3 to 8 times higher than the parent drug, while the persistent detection of 6-OH-CBD and 7-COOH-CBD at 6 h points to a prolonged elimination phase for those CBD metabolites.

Overall, traditional SLE data demonstrated a high absolute accumulation of total CBD across all brain regions sampled, while *ex vivo* SPME analysis revealed that the actual concentration of CBD was much lower, accounting for 2–40% of the total tissue burden. Nevertheless, future *in vivo* SPME applications require pre-equilibrium or negligible-depletion sampling regimes of analytes from brain regions. Thus, implementing smaller geometries of SPME probes (such as thinner coatings) or shorter extraction times (for diffusion-based calibration) alongside high-sensitivity MS detection will facilitate reliable analyte quantification while maintaining non-depleting conditions. Using total tissue SLE, the phase I metabolites, namely active 6-OH-CBD and 7-OH-CBD, and also inactive 7-COOH-CBD were successfully detected within each brain structure analyzed, confirming that these biotransformation products cross the BBB (Supplementary Table S4). However, the corresponding *ex vivo* SPME sampling recovered no free metabolites (except for above-mentioned one rat at 4 h time-point). The discrepancy between the high total tissue concentrations of 7-OH-CBD measured by SLE and its very low or undetectable recovery by SPME cannot be quantitatively attributed to tissue sequestration alone. Because SLE reflects the total tissue burden, whereas SPME preferentially samples the freely available fraction, the observed difference may result from extensive binding to tissue components, free concentrations below the SPME-LC-MS/MS detection limit (2.0 ng/g), or a combination of both. It should be noted that the 30-min static extraction period was optimized using a 4% low-temperature agarose gel to account for restricted analyte diffusion and mass-transfer resistance under semi-solid conditions; however, tissue-specific diffusion coefficients, tortuosity, and extraction kinetics were not determined for individual brain structures. Therefore, regional differences in mass transfer and coating-analyte interactions should be considered when interpreting the low or undetectable SPME recovery of phase I metabolites from individual brain regions. Therefore, the present data do not allow the relative contribution of these mechanisms to be quantitatively determined. While a larger animal cohort remain prerequisites to calculate absolute bioavailability values and population pharmacokinetic-pharmacodynamic parameters, these focused preliminary data successfully validate the technical feasibility of this multi-matrix microextraction workflow to monitor CBD levels and distribution across brain regions. This proof of concept establishes a robust experimental baseline, demonstrating that linking living intravascular extractions with localized regional brain sampling provides a resolution of active drug biophases in neuropharmacology.

## 4 Conclusion

This study establishes a novel, minimally invasive SPME sampling approach coupled with LC-MS/MS platform capable of monitoring the dynamic pharmacokinetics of unbound CBD, its primary metabolites, and key endocannabinoids across systemic circulation and discrete brain regions. By maintaining SPME-based extraction within intact biological matrices, this method overcomes the critical biophysical limitations of traditional tissue homogenization techniques. Crucially, this work reports for the first time the monitoring of CBD pharmacokinetics directly in the circulating blood of living organisms under *in vivo* conditions. The 10-min *in vivo* blood sampling successfully captured the systemic free fraction of parent CBD, revealing rapid oral absorption (Cmax ≈160 μg/mL at 2 h) followed by sharp systemic clearance driven by first-pass metabolism and rapid distribution into highly perfused lipophilic tissues. Simultaneously, this *in vivo* blood protocol provided real-time tracking of systemic endocannabinoid dynamics in living rats, with mean circulating free 2-AG concentrations numerically higher than baseline following oral CBD administration and reaching the highest mean value at 6 h post-dose. In addition to systemic observations, *ex vivo* microextraction of intact brain structures right after sacrifice revealed distinct spatial neuropharmacokinetics. While the amygdala and cerebellum exhibited peak free CBD levels at 2 h post-dosing, the hippocampus displayed a delayed peak at 4 h alongside the highest observed Cmax, a numerically greater AUC_0–6_ compared with the amygdala and cerebellum, and the highest numerical brain-to-serum AUC_0–6_ ratio. This delayed hippocampal CBD concentration profile coincided with numerically higher mean 2-AG concentrations at 4 and 6 h, with the highest mean value observed at 6 h. These findings indicate descriptive, region-dependent temporal patterns in 2-AG concentrations rather than statistically confirmed CBD-induced region-specific modulation of the ECS. While total tissue SLE detected high accumulation of phase I metabolites, with total 7-OH-CBD accumulating at levels 3 to 8 times higher than parent CBD, SPME preferentially extracts freely available concentrations of those analytes in brain structures. This difference may reflect extensive tissue association and/or freely available concentrations below the extraction or analytical detection capacity of SPME. This proof-of-concept study demonstrates that linking living intravascular *in vivo* sampling with localized regional brain microextraction provides insight into active biophase kinetics and highlights the potential value of free-fraction profiling for neuropharmacological evaluation and for investigating possible region-specific interactions between CBD exposure and the ECS.

## Data Availability

The raw data supporting the conclusions of this article will be made available by the authors, without undue reservation.
