# Supplementary material for: In vivo blood and ex vivo brain solid-phase microextraction coupled with LC-MS/MS for mapping unbound cannabidiol, phase I metabolites, and endocannabinoid tone
Source: Front Pharmacol. 2026 Oct 26;17:1980760. doi: 10.3389/fphar.2026.1980760 (PMC13745328; doi:10.3389/fphar.2026.1980760)
Supplement: Supplementary file 1 [file Supplementaryfile1.docx]

Supplementary Material

# 1 Supplementary Data

**1.1 Optimization of LC-MS/MS conditions**

Developing a unified analytical platform capable of simultaneously quantifying CBD, its phase I biotransformation products, and endocannabinoids presents a severe chromatographic and mass spectrometric challenge. The analytes under investigation span an exceptionally wide polar surface area and lipophilicity, ranging from the highly hydrophobic parent CBD and 2-AG (log P values greater than 6) to the highly polar, ionizable phase I metabolite 7-COOH-CBD. During instrumental optimization, standard gradient methods resulted in severe peak broadening for late-eluting lipophilic compounds or co-elution of the polar 7-COOH-CBD within the matrix suppression zone of the solvent void volume. Optimization of LC-MS/MS conditions concerned the selection of chromatographic column, mobile phase composition and gradient as well as MS working parameters (**Supplementary Tabel 1**). Three types of LC columns were tested, namely Poroshell 120 EC-C18 100 x 2.1 mm; 2.7 µm (Agilent, USA), Poroshell 120 PFP 100 x 2.1 mm, 2.7 µm (Agilent, USA), Kinetex XB-C18 100 x 2.1 mm, 2.6 µm (Phenomenex, USA). Based on the performed analyses, separation of all studied compounds was achieved on each column. The largest peak areas and the best peak shapes were obtained using EC-C18 column, while the smallest peak areas were observed during analyses with the Kinetex XB-C18 column. Also, different mobile phase compositions and ratios were tested, including ACN, MeOH, 0.1% FA, and 5 mM ammonium formate. Ultimately, phase A: H_2_O+0.1% FA, phase B: ACN + 0.1% FA were selected for further analysis. By accelerating the organic modifier (mobile phase B) from 40% to 95% within a tightly controlled window, we achieved sharp, symmetrical peak shapes for all target compounds within a single 10-minute runtime. A critical component of this optimization was achieving baseline chromatographic resolution between the structural isomers 7-OH-CBD and 6-OH-CBD. Because these compounds share identical precursor-to-product MS/MS fragmentation pathways (m/z 331.2/201.1), MS isolation alone was not sufficient as overlapping peaks would lead to severe over-quantification of the active 7-OH-CBD fraction. Hence, the MS operated in positive ion mode for 7-OH-CBD and in negative ion mode for 6-OH-CBD. Optimizing the column temperature to exactly 40°C combined with a low flow rate from 0.3 to 0.4 mL/min ensured complete baseline separation, guaranteeing absolute quantitative fidelity. This optimization allowed the method to achieve excellent linearity (R^2^ values exceeding 0.995) across all target compounds, eliminating the need for multiple sample dilutions.

**1.2 SPME method optimization**

Prior to quantitative analysis, optimization studies were performed in both surrogate matrices to evaluate the extraction kinetics of CBD and its metabolites and also selected endocannabinoids (AEA, NADA and 2-AG) onto the biocompatible SPME probes. For SPME method development, previously optimized parameters for extraction of endocannabinoids from brain samples, such as type of SPME coating, times of extraction and desorption, and desorption solvent were applied in this study (Roszkowska et al., 2023). For SPME blood sampling, a 50:50 (*v/v*) mixture of artificial serum and PBS was prepared and spiked with studied compounds and ISs. This liquid matrix was utilized to mimic the viscosity, ionic strength, and high protein-binding environment of circulating whole blood while maintaining analytical reproducibility during the in-lab optimization phase. For SPME brain sampling, matrix-matched calibration standards were validated using optimized surrogate matrices (agarose gel) to ensure accurate quantification and account for the distinct properties of the sampled compartments. A 4% low-temperature agarose gel (w/v) was prepared by dissolving low-melting-point agarose powder in PBS (pH 7.4) under gentle heating. The solution was poured into silanized glass vials, spiked with a mixture of analyzed compounds, and allowed to solidify at room temperature to create a semi-solid brain tissue phantom. This gel matrix was selected to replicate the mass transfer resistance and restricted analyte diffusion.

For optimization of SPME towards extraction of analytes from blood samples under *in vivo* conditions, C18 probes were used for 10 min static extraction from artificial serum, while the optimization of extraction of studied analytes from brain tissue was performed in low-temperature agarose gel under static conditions for 30 min. Two lengths of SPME coating were used, namely 10 mm coating was applied for *in vivo* blood sampling and also for *ex vivo* brainstem sampling (as this is relatively large brain structure), and 5 mm coating was applied for sampling of small brain structures, i.e. amygdala, cerebellum and hippocampus. As recently indicated, a reduced volume of extraction phase of SPME coating facilitate extraction of analytes from brain under negligible depletion conditions (<5%), thereby not disturbing the equilibrium between free and matrix-bound analyte forms within the analyzed matrix (Oliveira et al., 2026). The performed analysis also revealed that the type of SPME coating used in this study (C18 particles) was suitable for the extraction of all studied compounds, including CBD and its hydroxy-metabolites at low nanogram levels for both matrices. However, 7-COOH-CBD, a more polar CBD metabolite, could not be extracted in applied SPME conditions for *in vivo* sampling (10 min) at lower concentrations (< 50 ng/mL), but was successfully extracted at concentrations > 25 ng/g during 30 min static extraction from agarose gel. In theory, extending the extraction time to 30 min would allow the extraction of lower levels of this CBD metabolite from serum samples, however, due to ethical issues related to experiments performed on animals, such long extraction time was not allowed to be applied for the living organism, which is a main limitation of presented study.

**1.3 SLE-LC-MS/MS method validation**

Quantitative method validation comprises evaluation of linearity of proposed method. Matrix-matched calibration curves were constructed by spiking of surrogate matrix (homogenate of chicken breast) with working solutions of CBD and its metabolites. A calibration curve was 100-2000 ng/g for CBD, 10-1000 ng/g for both 7-OH-CBD and 6-OH-CBD, and 10-500 ng/g for 7-COOH-CBD. An IS (CBD-d_3_) was spiked at a final concentration (10 ng/g) to the surrogate matrix. Linearity was assessed by calculating the coefficient of determination (R^2^) using a weighted linear regression model (**Supplementary Table 3**).

**1.4 Brain sampling via SLE**

To provide a total mass benchmark for comparison against the SPME free-fraction datasets, the remaining tissue from all dissected brain regions (hippocampus, amygdala, cerebellum, brainstem) collected from 1, 2, 4 and 6 hr time-points after CBD administration and from control group was subjected to exhaustive SLE dilutions (**Supplementary Table 4**). After SPME sampling was completed, the brain was dissected into selected structures that were placed in Eppendorf tubes and stored at –80°C until SLE procedure according to the Folch method with modifications (Folch et al., 1957; Bystrowska et al., 2014; Smaga et al., 2017). On the day of the experiment, tissues were weighed and homogenized in ice-cold methanol containing fixed concentrations of the ISs (AEA-d_11_ and CBD-d_3_) at final concentration of 10 ng/g of tissue using a tissue glass grinder pestle Kimble (Merck, Germany) at a ratio of 10 µL MeOH per 1 mg of tissue. Next, 750 µL of MeOH:chloroform mixture (500:250, *v/v*) was added to the homogenates and vortexed for 10 sec. Samples were placed in the fridge for 10 min, and then the supernatant was collected into a new 15 mL corning tube. Another 750 ul of MeOH:chloroform mixture (500:250, *v/v*) was added to the homogenate and vortexed for 10 sec. Samples were placed in the fridge for 10 min, and then the supernatant was collected and added to the previously collected fraction. Samples were centrifuged (3400 rpm, 10 min, 4°C) (VWR Mega Star 600R, VWR International, Germany), and the supernatants were collected and evaporated to dryness at 30°C (Concentrator plus, Eppendorf, Germany), and dry residue was stored at –80°C until reconstitution prior to LC-MS/MS analysis.

# 2 Supplementary Figures and Tables

## 2.1 Supplementary Figures

**Figure S1.** The impact of the temperature of sample matrix on the efficiency of SPME extraction efficiency for selected analytes.

**Figure S2.** Time-dependent changes in CBD concentration in the **(A)** amygdala, **(B)** cerebellum, and **(C)** hippocampus following CBD administration. Data are presented as mean ± SD; each value represents the mean, n = 2–3 rat per groups. Statistical analysis was performed using the Kruskal–Wallis test followed by Dunn's multiple comparisons test *p < 0.05

**Figure S3.** Time-dependent changes in 2-AG concentration in the (**A**) amygdala, (**B**) cerebellum, and (**C**) hippocampus following CBD administration. Data are presented as mean ± SD; ach value represents the mean, n = 2–3 values per groups. Statistical analysis was performed using the Kruskal–Wallis test followed by Dunn's multiple comparisons test. No significant differences.

## 2.2 Supplementary Tables

**Supplementary Table 1**. Optimized MS parameters for target precursor/product ion transitions, collision energy (CE), and declustering potential (DP) and retention time (rt) for each analyte.

| Compound | Precursor ion  *m/z* | Product ions  *m/z* | CE  (V) | DP  (V) | rt  (min) |
| --- | --- | --- | --- | --- | --- |
| CBD | 315.15 | 193.12  123.05 | 33.0  48.0 | 75.0  75.0 | 6.78 |
| 7-OH-CBD | 331.23 | 313.22  193.12 | 17  28 | 65.0  65.0 | 6.13 |
| 6-OH-CBD | 329.12 | 311.23  173.08 | -23  -34 | -110.0  -100.0 | 6.08 |
| 7-COOH-CBD | 345.18 | 327.19  299.22 | 22  27 | 90.0  90.0 | 6.06 |
| CBD-d_3_ | 318.29 | 196.15  123.06 | 30  46 | 70.0  70.0 | 6.78 |
| 2-AG | 379.29 | 287.27  269.26 | 21  24 | 85.0  85.0 | 5.2 |
| AEA | 348.30 | 330.30  287.30 | 16  16 | 60.0  60.0 | 5.00 |
| NADA | 440.34 | 137.06  154.09 | 42  23 | 80.0  80.0 | 5.10 |
| AEA-d_11_ | 359.36 | 91.04  298.28 | 65  16 | 60.0  60.0 | 5.00 |

**Supplementary Table 2.** Mean CBD (µg/ml) and 2-AG (ng/ml) concentrations in rat blood following oral administration of a single dose of CBD (100mg/kg).

| **Mean** | **Time (h)** | | | | |
| --- | --- | --- | --- | --- | --- |
|  | **Control** | **1** | **2** | **4** | **6** |
| CBD | ND | 5.6 | 164.29 | 2.0 | 4.57 |
| 2-AG | 34.45 | 95.89 | 93.80 | 103.81 | 180.48 |

ND – not detected; each value represents the mean, n = 2–3 rat per groups.

**Supplementary Table 3**. Linearity parameters for CBD and its metabolites in brain samples using SLE-LC-MS/MS method.

| analyte | range (ng/g) | linear regression | R^2^ |
| --- | --- | --- | --- |
| CBD | 100-2000 | y = 0.0016x + 0,7075 | 0.969 |
| 7-OH-CBD | 10-1000 | y = 0.007x + 0,09 | 0,9836 |
| 6-OH-CBD | 10-1000 | y = 0.0031x - 0,0497 | 0.9984 |
| 7-COOH-CBD | 10-500 | y = 0.0083x - 0,0269 | 0.9997 |

**Supplementary Table 4**. Mean concentrations (µg/g) of CBD and its metabolites in rat brain over 6 hr time course following oral administration of a single dose of CBD (100 mg/kg). Analyses were performed via SLE-LC-MS/MS method.

|  | 1 hr | 2 hr | 4 hr | 6 hr |
| --- | --- | --- | --- | --- |
| CBD | | | | |
| amygdala | 0.46±0.14 | 4.441±0.06 | 4.396±0.86 | 0.32±0.20 |
| hippocampus | 0.258±0.09 | 1.899±0.04 | 0.851±0.06 | 0.11±0.09 |
| cerebellum | 0.120±0.03 | 1.272±0.21 | 2.260±0.01 | 0.29±0.13 |
| brain stem | 0.31±0.11 | 3.086±0.29 | 2.537±0.78 | 0.18±0.07 |
| 7-OH-CBD | | | | |
| amygdala | 0,927±0,49 | 2,976±0,72 | 16,012±7,48 | nd |
| hippocampus | 1,829±1,11 | 5,726±1,13 | 17,790±0,95 | nd |
| cerebellum | 2,129±1,26 | 7,444±1,53 | 16,482±5,67 | 0,080±0,04 |
| brain stem | 1,718±0,98 | 8,574±2,51 | 22,686±4,28 | nd |
| 6-OH-CBD | | | | |
| amygdala | 0,633 | 2,195±1,03 | 1,670±0,46 | 0,175±0,03 |
| hippocampus | 0,659±0,29 | 1,403±0,11 | 1,034±0,01 | 0,177±0,14 |
| cerebellum | 0,550±0,07 | 1,317±0,06 | 1,058±0,61 | 0,179±0,10 |
| brain stem | 0,642±0,03 | 1,104±0,13 | 1,268±0,35 | 0,399±0,34 |
| 7-COOH-CBD | | | | |
| amygdala | 0,157±0,05 | 0,416±0,25 | 0,479±0,22 | 0,240±0,09 |
| hippocampus | 0,290±0,14 | 0,642±0,34 | 0,368±0,29 | nd |
| cerebellum | 0,282±0,10 | 0,463±0,10 | 0,472±0,34 | 0,048±0,02 |
| brain stem | 0,263±0,11 | 0,248±0,08 | 0,394±0,32 | nd |

nd – not detectable

# 3 References

Bystrowska, B., Smaga, I., Tyszka-Czochara, M., and Filip, M. (2014). Troubleshooting in LC-MS/MS method for determining endocannabinoid and endocannabinoid-like molecules in rat brain structures applied to assessing the brain endocannabinoid/endovanilloid system significance. *Toxicol. Mech. Methods* 24, 315–322. doi: 10.3109/15376516.2014.898356

Folch, J., Lees, M., and Sloane Stanley, G. H. (1957). A simple method for the isolation and purification of total lipides from animal tissues. *J. Biol. Chem.* 226, 497–509.

Oliveira, I. G. C., Nascimento, G. C., Del-Bel, E., Pawliszyn, J., and Queiroz, M. E. C. (2026). *In Vivo* Negligible Depletion SPME for the Determination of Free and Total Concentrations of Anandamide and 2-Arachidonoylglycerol in the Brain of a Parkinson’s Disease Rat Model. *Anal. Chem.* 98, 12484–12497. doi: 10.1021/acs.analchem.5c07902

Roszkowska, A., Klejbor, I., Bogusiewicz, J., Plenis, A., Bojko, B., Kowalik, K., et al. (2023). Monitoring of age- and gender-related alterations of endocannabinoid levels in selected brain regions with the use of SPME probes. *Metabolomics* 19, 40. doi: 10.1007/s11306-023-02007-9

Smaga, I., Jastrzębska, J., Zaniewska, M., Bystrowska, B., Gawliński, D., Faron-Górecka, A., et al. (2017). Changes in the Brain Endocannabinoid System in Rat Models of Depression. *Neurotox. Res.* 31, 421–435. doi: 10.1007/s12640-017-9708-y
